# Deep Fungal Infections Among General Hospital Inpatients in Southwestern China: A 5-Year Retrospective Study

**DOI:** 10.3389/fpubh.2022.842434

**Published:** 2022-03-28

**Authors:** Shu-Ran Wen, Zheng-Hui Yang, Tian-Xiang Dong, Yu-Ye Li, Ying-Kui Cao, Yi-Qun Kuang, Hong-Bin Li

**Affiliations:** ^1^Department of Dermatology and Venereology, First Affiliated Hospital of Kunming Medical University, Kunming, China; ^2^NHC Key Laboratory of Drug Addiction Medicine, First Affiliated Hospital of Kunming Medical University, Kunming Medical University, Kunming, China; ^3^Scientific Research Laboratory Center, First Affiliated Hospital of Kunming Medical University, Kunming, China

**Keywords:** deep fungal infections, epidemiology, China, general hospital, retrospective study

## Abstract

**Background:**

Deep fungal infection is a type of life-threatening opportunistic infection. Its incidence has been increasing in recent years. This infection can affect the prognosis of patients, prolong hospital stays and raise costs for patients and their families.

**Objective:**

We aimed to understand the current situation of deep fungal infections in the First Affiliated Hospital of Kunming Medical University and to provide a basis for the clinical diagnosis and treatment of deep fungal infections.

**Methods:**

This was a retrospective analysis of 528,743 cases in the hospital from 2015 to 2019, including the epidemiological characteristics, treatment and prognosis of deep fungal infections.

**Results:**

A total of 274 cases (0.05%) with deep fungal infections were identified, accounting for 0.05% of the total number of hospitalizations. The incidence of deep fungal infections in the hospital showed an increasing trend from 2015 to 2019. The most commonly infected site was the respiratory tract (93.07%). Among patients with deep fungal infections, 266 specimens were positive for fungal culture, by which 161 cultured *Candida albicans* (*C. albicans*), accounting for 60.53%, the main pathogen causing deep fungal infection. From 2015 to 2019, the percentage of *C. albicans* cases showed a downward trend, while that of non-*C. albicans* showed an opposite trend. Antibiotics were the most common predisposing factor for deep fungal infections (97.45%). Among the underlying diseases of patients with deep fungal infections, infectious diseases (59.49%) were the most common. Those with underlying diseases such as renal insufficiency and neurological diseases had a worse prognosis. Indwelling catheters, nervous system disease and tumors were risk factors for a poor prognosis.

**Conclusions:**

We report for the first time the epidemiological data of deep fungal infections in a general hospital in southwestern China from 2015 to 2019. In the past 5 years, the number of patients with deep fungal infections in the First Affiliated Hospital of Kunming Medical University has been increasing. Although the clinical data are limited, these results can provide references for the diagnosis and treatment of deep fungal infections.

## Introduction

Deep fungal infections can involve subcutaneous tissue, mucosa, and internal organs, including limited single-organ infections and systemic infections involving multiple organs. Deep fungal infection is a life-threatening opportunistic infection that commonly occurs in immunocompromised individuals, and its incidence has been increasing in recent years (1). The occurrence of deep fungal infections is related to the widespread use of broad-spectrum antibacterial drugs, immunosuppressive agents, catheter technology, and organ transplantation, and the increase in patients with AIDS, malignant tumors, and the elderly (2).

Epidemiological research reports showed that among patients who died of deep fungal infection in general hospitals, the mortality rate caused by candidiasis was 50–71% (1). Candida is the most common pathogen of deep fungal infections (3). It is widely found in human skin and mucous membranes and is usually maintained as a benign colonization state.

At least 15 different Candida species have been identified to be associated with diseases in humans. More than 90% of invasive candidiasis cases are caused by the five most common pathogens: *Candida albicans (C. albicans), Candida glabrata (C. glabrata), Candida tropicalis (C. tropicalis), Candida parapsilosis (C. parapsilosis)*, and *Candida krusei (C. krusei*) (4). Although *C. albicans* is still the most common species, the infection rate of non-*C. albicans* spp. has increased significantly in recent decades, especially in ICU patients (5–7). According to an epidemiological study conducted in Europe, the mortality rate of patients with non-*C.albicans* infections is higher than that of patients with *C. albicans* infections (47.3 and 32.4%, respectively) (8, 9). Bloodstream infections caused by non-*C. albicans* spp. are difficult to treat due to antifungal drug resistance and have a high mortality rate (10–14).

The common pathogens of deep fungal infections are *Aspergillus* and *Cryptococcus* rank after Candida (3). In recent years, some rare filamentous fungi such as *Mucor* and *Fusarium* have been reported from time to time in patients with hematological malignancies and bone marrow transplantation (11). In this study, we conducted a retrospective analysis of the status of deep fungal infections in the First Affiliated Hospital of Kunming Medical University from January 2015 to December 2019.

## Materials and Methods

### Patient Selection

We screened inpatients from January 2015 to December 2019 from the medical record room of the First Affiliated Hospital of Kunming Medical University, and then screened out cases of fungal infection by their laboratory tests. Each hospitalization represented a case, and if the patient was hospitalized again and received another round of treatment, it was considered a new independent case. The same patient was considered to be a case if the same fungus was cultured multiple times from specimens from the same source during the same hospitalization period. Case exclusion criteria: cases with incomplete data on epidemiology, fungal microscopy, and culture examinations.

### Criteria for Study Inclusion

A retrospective investigation method was used to retrospectively analyse 528,743 medical records from the inpatient department of the First Affiliated Hospital of Kunming Medical University from January 2015 to December 2019 and perform statistical analysis on the cases of fungal infection by laboratory tests. The diagnostic criteria for patients with deep fungal infections included in this study were based on the diagnostic criteria for nosocomial infections, EORTC/MSGERC (12), and the diagnostic criteria for deep fungal infections formulated by the Chinese Journal of Internal Medicine (13). The information analyzed included general information about the patient, related information about the underlying disease, fungal infection, and information about the use of antifungal drugs.

### Methods for Identifying Fungal Agents

Fungal identification was done by fungal direct microscopy and mycologic culture. The samples such as sputum, cerebrospinal fluid, blood, urine, exudates were inoculated on Sabouraud's dextrose agar with chloramphenicol at 35°C for 5–7 days. Positive samples were sub-cultured on Sabouraud chloramphenicol agar or blood agar at 35°C for 24–48 h. The yeasts were identified by API 20 C AUX, other filamentous fungi were identified by biochemical and morphological features.

### Statistical Analysis

Statistical analysis was performed using SPSS 25.0. This study is based on the count data described by the number of cases and the percentage. The test chi-square test was used for comparisons between groups of enumeration data. A two-tailed *t*-test was used to determine significant differences between the means in age and the length of stay. Binary logistic regression was used to compare multivariate associations. *P* < 0.05 indicates that the difference is statistically significant.

## Results

### Incidence and Annual Distribution of Deep Fungal Infections

**Figure 1:**
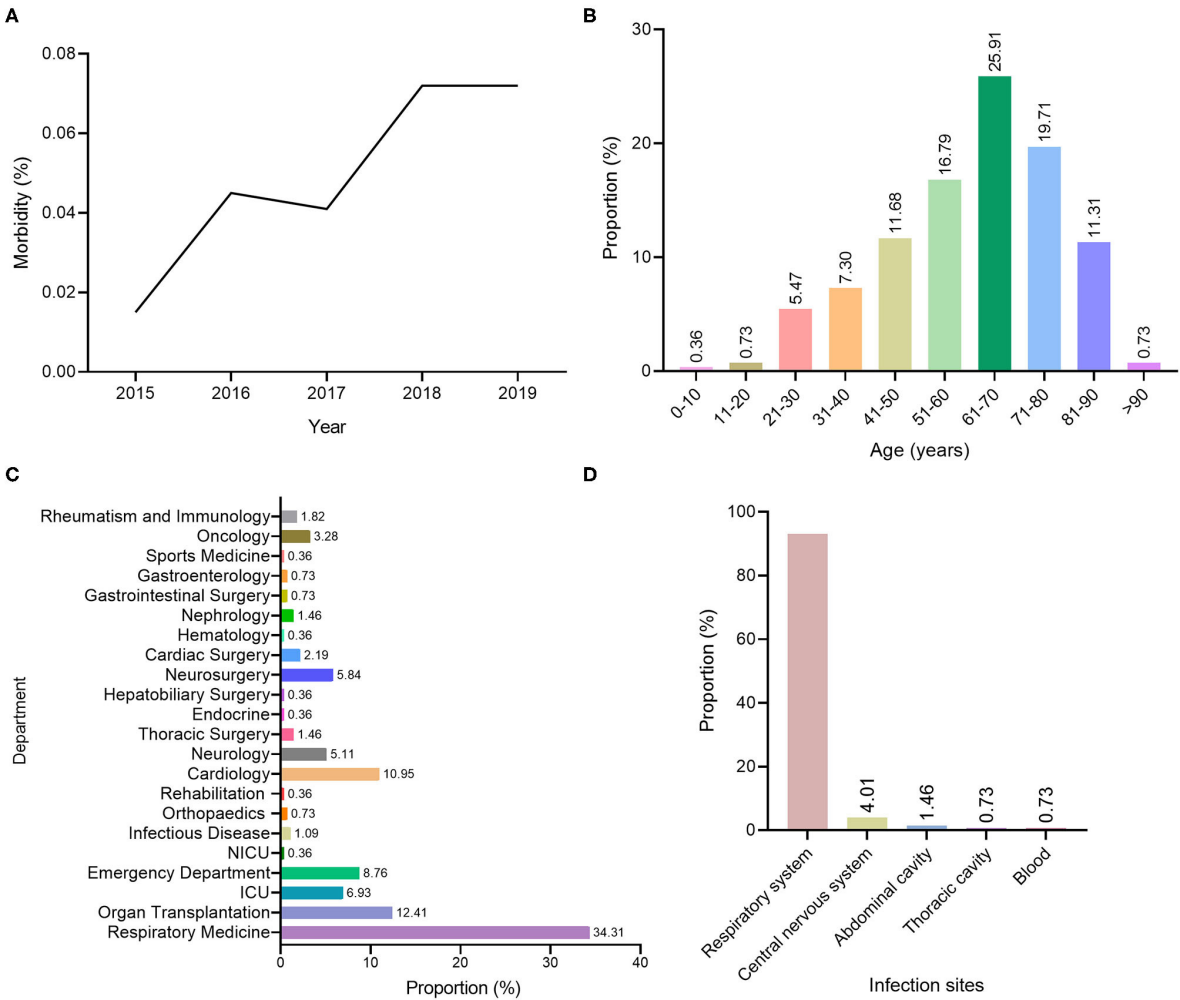
Distribution of selected characteristics of deep fungal infections. **(A)** Annual distribution of the rate of deep fungal infections. **(B)** Distribution of patients with deep fungal infections in different age groups. **(C)** Distribution diagram of departments with patients with deep fungal infections. **(D)** Distribution of deep fungal infection sites.

From January 2015 to December 2019, there were 528,743 inpatients in the First Affiliated Hospital of Kunming Medical University, including 86,551, 88,393, 105,890, 119,575 and 128,334, respectively. According to the above diagnostic criteria, 274 (0.05%) cases of deep fungal infections were diagnosed. The diagnosed deep fungal infection case numbers and the rates (the number of diagnosed cases of deep fungal infection/the total number of hospitalized cases in the year × 100%) were 13 (0.015%), 40 (0.045%), 43 (0.041%), 86 (0.072%), and 92 (0.072%) from 2015 to 2019, respectively. Retrospective analysis of 528,743 cases demonstrated that the incidence of deep fungal infections showed an increasing trend from January 2015 to December 2019, during which 2018 and 2019 had the highest incidences, whose highest values were the same (0.072%) (Figure 1A).

### General Situation of Patients With Deep Fungal Infections

The sex statistics of patients diagnosed with deep fungal infections were 187 men, accounting for 68.25%, and 87 women, accounting for 31.75% (Table 1). The age of the patients with deep fungal infections ranged from 4 years old to 93 years old, with an average age of 61.04 ± 16.89. Most of them were distributed in the three age groups of 51–60 years old (16.79%), 61–70 years old (25.91%), and 71–80 years old (19.71%). Patients over 60 years old accounted for 57.66% of the total number of deep fungal infections (Figure 1B).

**Table 1:** Demographic data of patients with deep fungal infections.

**Characteristic**	**Case number**
Gender	
Male	187 (68.25%)
Female	87 (31.75%)
Average age (years)	61.04 ± 16.89
Length of stay (days)	26.16 ± 26.63

The top three departments diagnosing patients with deep fungal infections were the respiratory department (94 cases, 34.31%), the transplantation department (34 cases, 12.41%), and the cardiology department (30 cases, 10.95%) (Figure 1C).

The main site of deep fungal infection was the respiratory system with 255 cases (93.07%), followed by central nervous system with 11 cases(4.01%), the abdominal cavity with 4 cases (1.46%) and the thoracic cavity and blood with 2 cases each (0.73%) (Figure 1D).

### Pathogens of Deep Fungal Infections

**Figure 2:**
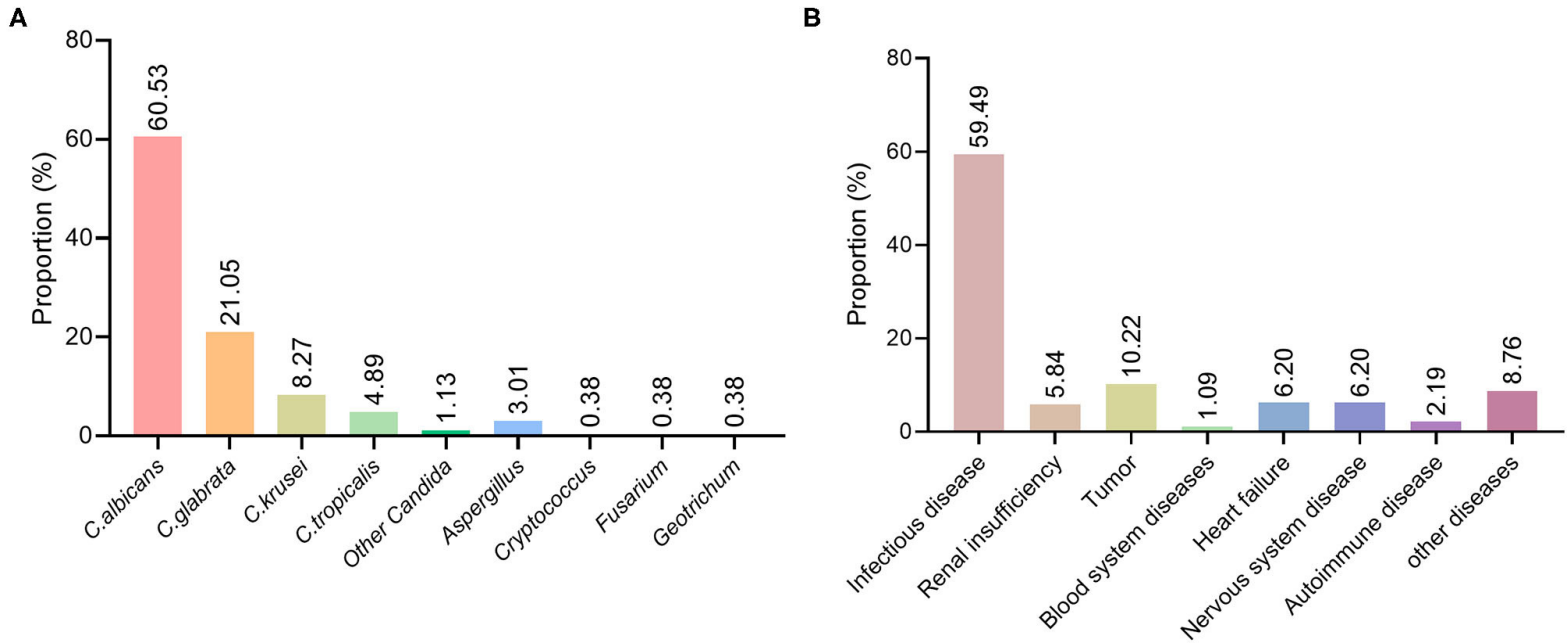
Distribution of pathogenic fungi and basic diseases in patients with deep fungal infections. **(A)** Distribution of various fungal species in patients with deep fungal infections. **(B)** Distribution of underlying diseases in patients with deep fungal infections.

Among the patients with deep fungal infections, 266 specimens were positive on fungal culture. The results were as follows: 161 cases (60.53%) of *C. albicans*, 56 cases (21.05%) of *C. glabrata*, 22 cases (8.27%) of *C. krusei*, 13 cases (4.89%) of *C. tropicalis*, 3 cases (1.13%) of other Candida, 8 cases (3.01%) of *Aspergillus*, 1 case (0.38%) each of *Cryptococcus, Fusarium*, and *Geotrichum* (Figure 2A). From 2015 to 2019, the percentage of *Candida albicans* showed a downwards trend, while the percentage of non-*C. albicans* spp. showed an upwards trend.

### Predisposing Factors of Patients With Deep Fungal Infections

Among the 274 patients diagnosed with deep fungal infections, 267 (97.45%) had history of using antibiotics, 116 (42.34%) had history of using indwelling catheters, 113 (41.24%) had history of using glucocorticoids, 31 (11.31%) had history of using immunosuppressive agents, 1 (0.36%) had history of using chemotherapy drugs, and 3 (1.09%) had no specific predisposing factors (Table 2). Indwelling catheters were a risk factor for a poor prognosis (OR = 3.525, 2.041-6.089).

**Table 2:** Percentage of risk factors in patients with deep fungal infections.

**Risk factors**	**Case of deep fungal infections (*n*, %)**
Antibiotic	267 (97.45%)
Glucocorticoid	113 (41.24%)
Immunosuppressant	31 (11.31%)
Indwelling catheter	116 (42.34%)
Chemotherapeutic drugs	1 (0.36%)
No susceptibility factors	3 (1.09%)

Among the 274 patients diagnosed with a deep fungal infection, 90 patients (32.85%) had 1 predisposing factor, 122 patients (44.53%) had 2 predisposing factors, and 42 patients had 3 predisposing factors. (15.33%), while 17 cases (6.20%) had 4 predisposing factors.

### Analysis of Underlying Diseases in Patients With Deep Fungal Infections

Among the 274 patients diagnosed with deep fungal infections, 163 had an underlying infectious disease, including an acute exacerbation of chronic obstructive pulmonary disease, pyelonephritis, central nervous system infection, pneumonia, AIDS, tuberculosis, and cholecystitis, accounting for 59.49%;16 cases of renal insufficiency, accounting for 5.84%; 28 cases of tumors, accounting for 10.22%; 3 cases of hematological diseases, accounting for 1.09%; 17 cases of cardiac insufficiency, accounting for 6.20%; 17 cases of neurological diseases, accounting for 6.20%; 6 cases of autoimmune diseases, accounting for 2.19%; and 24 cases of other diseases, accounting for 8.76% (Figure 2B).

### The Use of Antifungal Drugs for Deep Fungal Infections

**Figure 3:**
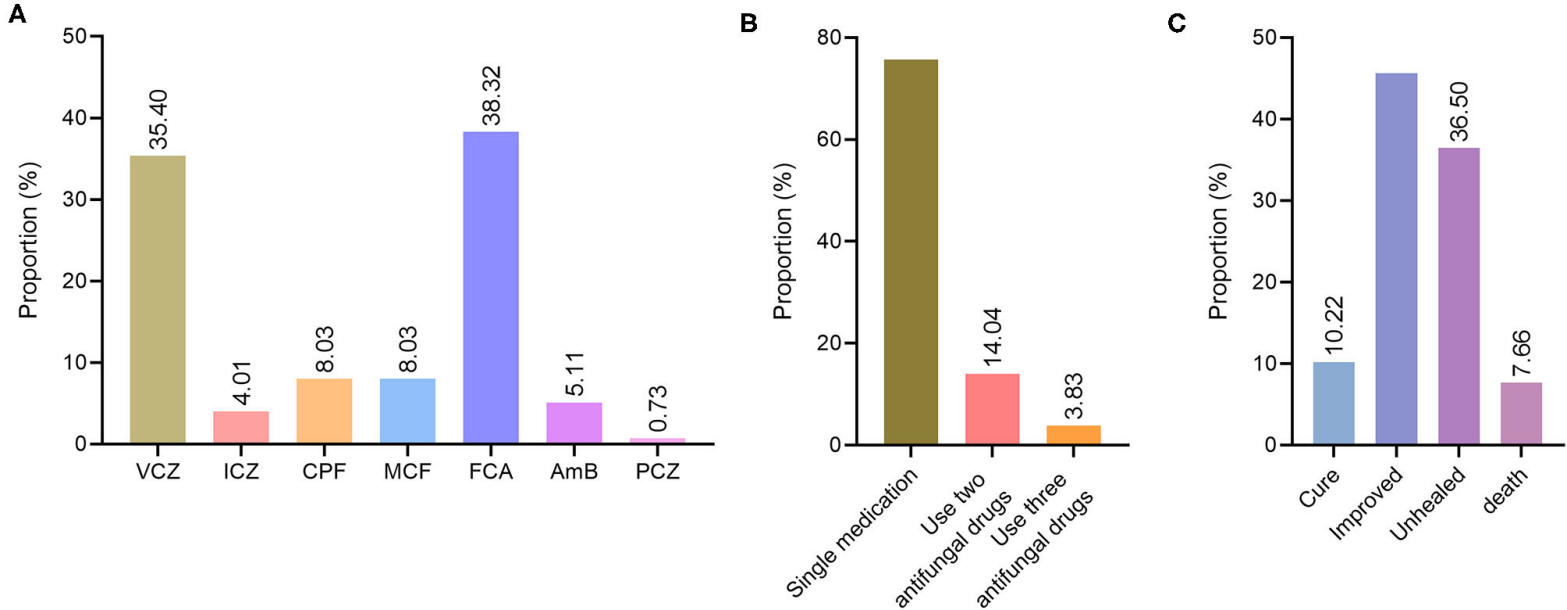
Treatment and prognosis of deep fungal infections. **(A)** Distribution diagram of antifungal drug species for deep fungal infections. VCZ, voriconazole; ICZ, itraconazole; CPF, caspofungin; MCF, micafungin; FCA, fluconazole; AmB, amphotericin B; PCZ, posaconazole. **(B)** Distribution of antifungal drug use patterns in patients with deep fungal infections. **(C)** The distribution of prognostic types for deep fungal infections.

Of the 274 patients with deep fungal infections, 220 were treated with antifungal drugs, accounting for 80.29%: 97 cases of voriconazole (35.40%), 11 cases of itraconazole (4.01%), 22 cases of caspofungin (8.03%), 22 cases of micafungin (8.03%), 105 cases of fluconazole (38.32%), 14 cases of amphotericin B (5.11%) and 2 cases of posaconazole (0.73%) (Figure 3A).

Among the 220 patients with deep fungal infections who used antifungal drugs, 178 were single drugs, accounting for 75.74%, and 42 (17.87%) were used in combination, alternate use, or combined/alternative use. Among them, two antifungal drugs were used in 33 cases (14.04%), and three antifungal drugs- were used in 9 cases (3.83%) (Figure 3B).

The prognosis of 153 (55.84%) patients among the 274 with deep fungal infections was good (cured + improved), including 28 cured (10.22%) and 125 improved (45.62%). A poor prognosis (unhealed + death) was observed for 121 (44.16%) of the deep fungal infections, including unhealed 100 cases (36.50%) and 21 died cases (7.66%) (Figure 3C).

### Analysis of Factors Related to a Poor Prognosis of Patients With Fungal Infections

There was no statistically significant difference in age, sex or length of hospital stay between patients with deep fungal infections with a good prognosis and those with a poor prognosis (*P* > 0.05). Among the basic diseases, patients with deep fungal infections with renal insufficiency, tumors, and neurological diseases had a poor prognosis (*P* < 0.05). A tumor was a risk factor for a poor prognosis (OR = 2.558, 1.127–5.804). A nervous system disease was also a risk factor for a poor prognosis (OR = 5.288, 1.620–17.265). See Table 3 for details.

**Table 3:** Comparison of different prognostic related factors in patients with deep fungal infections.

	**Case number**	**Favorable prognosis**	**Poor prognosis**	***X^**2**^/t***	***P* value**
Gender					
Male	187	100 (53.48%)	87 (46.52%)	0.952	0.329
Female	87	52 (59.77%)	35 (40.23%)		
Average age		61.14 ± 16.56	60.92 ± 17.36	0.107	0.915
Length of stay (days)		27.15 ± 25.10	24.92 ± 28.51	0.689	0.492
Basic disease					
Infectious disease	163	95 (58.3%)	68 (41.7%)	0.974	0.324
Renal insufficiency	16	14 (87.5%)	2 (12.5%)	6.907	0.009
Tumor	28	10 (37.0%)	18 (63.0%)	4.294	0.038
Heart failure	17	8 (47.1%)	9 (52.9%)	0.567	0.452
Nervous system disease	18	4 (22.2%)	14 (77.8%)	8.829	0.003
Other	33	22 (66.67%)	11 (33.33%)	1.784	0.182
Types of antifungal drugs					
Azoles	189	131 (69.31%)	58 (30.69%)	44.847	<0.001
Echinocandins	41	31 (75.61%)	10 (24.39%)	7.642	0.006
Amphotericin B	14	5 (35.71%)	9 (64.29%)	2.423	0.120
Medication				101.650	<0.001
No antifungal drugs	54	0	54		
Single medication	178	126	52		
Two or more antifungal drugs	42	27	15		

## Discussion

As a life-threatening opportunistic infection, the incidence of deep fungal infection has increased in recent years (1, 14, 15). Recent studies have shown that 1.5 million people die from fungal infections worldwide each year, which is similar to the number of deaths caused by tuberculosis each year (16). The lack of specific symptoms makes the diagnosis of deep fungal infections more difficult. Once inpatients develop a deep fungal infection, it will increase the patient's hospital stay and economic costs. Therefore, research and analysis into deep fungal infections in general hospitals are of significance for the clinical diagnosis and treatment of deep fungal infections and for improving the prognosis of the patients.

This study demonstrated that from 2015 to 2019, deep fungal infections in the First Affiliated Hospital of Kunming Medical University showed an upward trend in prevalence from year to year, and reaching a peak in 2018 and 2019. According to the literature, fungal diseases are becoming more predominant in the United States, with 1,047,422 deaths in 2018 (17). Our research results are consistent with those reported in the previous literature. In recent years, the incidence of deep fungal infections has been increasing. In addition to the increased use of antibiotics, immunosuppressants, and indwelling catheters, this may also be related to the improvement of diagnostics. Improved fungal tests helps to identify patients with fungal infections earlier and enables timely intervention and treatment so that patients can obtain a better outcome (18).

Many studies have shown that age is a risk factor for deep fungal infections (1). Most elderly patients have a variety of underlying diseases, low immune function, and long hospital stays, and they are prone to multisystem infections. At the same time, their defense ability of the skin and mucous membranes is reduced, so their risk of fungal infection is greatly increased. In this study, elderly patients with deep fungal infections accounted for 57.66% of patients diagnosed with deep fungal infections. With the aging of the population in China and the increase in elderly patients, clinicians should pay attention to the higher risk of deep fungal infections in elderly patients and strive to improve the immunity of elderly patients.

Previous studies have demonstrated that fungal infections mainly occur in the respiratory system and urinary system (19). The analysis of this study showed that patients with deep fungal infections mainly came from the respiratory department and transplantation department, accounting for 34.31 and 12.41% respectively. The main sites of infection were the respiratory system, central nervous system and abdominal cavity, which accounted for 93.07, 4.01, and 1.46% respectively. During clinical treatment, special attention should be given to patients in departments with a high incidence of respiratory fungal infections, especially respiratory and transplantation departments. The reason why deep fungal infections occurred more frequently in the respiratory department may be that patients in the respiratory department were hospitalized for a long time. Prolonged hospitalizations may increase the exposure of fungal pathogens. Fungal pathogens are very abundant in hospitals settings and difficult to eradicate (20). In addition, most of the patients in the respiratory department were elderly patients with serious underlying diseases. Most of them needed to use broad-spectrum antibiotics. For elderly patients, the ability to clear cilia of respiratory tract mucosa is weak, and there is more hyperplasia and secretion by bronchial glands (21), and reduced pathogen clearance ability. There are many normal microorganisms colonizing the respiratory tract. Only when the immune function of the body is weakened can these opportunistic pathogens cause infection in the corresponding site. In addition, the large number of patients with deep fungal infections in the respiratory department may be correlated with the importance attached to fungal infections by respiratory doctors in our hospital so that relevant examinations are performed in a timely fashion to confirm the diagnosis. At the same time, due to the development of transplantation technology in recent years, the increase in transplant patients, invasive surgery and the widespread use of antibiotics, hormones or immunosuppressive agents, there is an increased susceptibility to deep fungal diseases, so the transplantation department has become a high incidence department of deep fungal infections.

Candida infections are ranked first in the species distribution of deep fungal infections. Among them, *C. albicans* was the main pathogen causing deep fungal infections (60.53%), followed by *C. glabrata* (21.05%). This result is consistent with the distribution of fungal species reported in the literature (22). The proportion of *C. glabrata* in *Candida* infections have been increasing in recent years. According to international reports, *C. glabrata* accounts for 17%-26% of patients with invasive fungal infections (23, 24). Our results are consistent with this finding. *C. glabrata* is resistant to a variety of antifungal drugs, and its invasive infection has a fatality rate of up to 50%, which brings great difficulties to the treatment of patients with invasive fungal infections (25). Most *C. glabrata* are naturally resistant to azole antifungal drugs (26). The mortality rate of patients with *C. glabrata* candidaemia who used voriconazole was significantly higher than that of patients who used echinocandin (27). The wrong choice of azole antifungal drugs by clinicians may lead to a further decrease in the patient's immunity, which means they are more likely to have multiple Candida infections. Other studies have suggested that invasive *C. glabrata* infection is likely to occur in patients with weakened immunity and neutropenia (25), which may be the reason why patients with invasive *C. glabrata* infection are more likely to have multiple Candida infections. According to previous reports, in general hospitals in northeastern China, the most common pathogen of deep infections is *C. parapsilosis* (34.8%), followed by *C. gillimonella* (26.7%), *C. albicans* (18.5%), and *C. glabrata* (8.1%) (20). This is different from the findings of this study, which may be due to differences in geographic regions, climate and temperature. The literature reports that 69.2% of aspergillosis occurs in the ICU (28). Our research identified aspergillus as accounting for 3.01% of deep fungal infections. This relatively low incidence may be correlated with the relatively dry climate in this region.

The use of broad-spectrum antibiotics, glucocorticoids, immunosuppressants, indwelling catheters, and chemotherapy drugs are common predisposing factors for deep fungal infections. Our study found that the use of antibiotics was the most prominent predisposing factor for deep fungal infections (97.45%), which is consistent with previous report (29). This was followed by indwelling catheters (42.34%)and the long-term use of glucocorticoids (41.24%). For the application of broad-spectrum antibiotics, the wider the scope of the antibiotic, the stronger its antibacterial ability, and the greater the risk that it will cause fungal infections, especially carbapenems, third-generation cephalosporins and fourth-generation quinolones. Furthermore, our study found that the most commonly used antifungal drugs in our hospital for the treatment of deep fungal infections were voriconazole, itraconazole, caspofungin, micafungin, fluconazole, amphotericin B, and posaconazole, among which the top two ranking drugs were fluconazole (38.32%) and voriconazole (35.40%) both of which are triazoles. Most of the deep fungal infections in our hospital were treated with single medications, which is consistent with the trend of previous research results. Clinically, patients with mild symptoms are mostly treated with a single medication, while severe deep fungal infections are often treated with combination medications.

Indwelling catheters can increase the risk of fungal infections. Candida is currently the leading cause of bloodstream infections with high mortality and high morbidity (30). Candidemia is highly related to the formation of biofilms on a central venous catheter. Catheters can provide an attachment surface for Candida, allowing it to settle and form a biofilm, while enhancing its resistance to antifungal drugs, especially azoles and polyenes, thereby promoting the formation of infections (30, 31). The biofilm formed by Candida is more resistant to antifungal drugs (31). Therefore, the formation and development of biofilms on medical indwelling catheters is an ongoing problem during clinical treatment (32). Clinically, patients with indwelling catheters should be alert to increased risk of fungal infections.

Infectious diseases (59.49%) and tumors (10.22%) are the most important underlying diseases in patients with deep fungal infections. Infections and tumors damage the local or overall immune function of the body so that the fungus can break through the immune defense of the body and cause deep tissue infection or fungal bacteremia. This study found that among the underlying diseases, renal insufficiency, tumors, and neurological diseases can influence the prognosis of deep fungal infections (*P* < 0.05). Due to the lack of specific clinical manifestations of deep fungal infection, infectious diseases such as HIV, tuberculosis, lung bacterial infection, bacteremia, etc. not only increase the difficulty of diagnosis of a deep fungal infection but also aggravate the patient's condition (33).

Most patients with deep fungal infections had a good prognosis (55.84%), and their age did not affect their prognosis, while underlying diseases such as renal insufficiency, tumors, and neurological diseases affected the prognosis of deep fungal infection (*P* < 0.05). Therefore, for patients with underlying diseases, especially renal insufficiency, tumors, and neurological diseases, attention should be given to the possibility of deep fungal infections, and the use of antifungal drugs should be emphasized to improve their prognosis and avoid deterioration of their disease.

## Conclusions

From 2015 to 2019, the deep fungal infection rate and *Candida glabrata* infections in the First Affiliated Hospital of Kunming Medical University both showed an upwards trend. Deep fungal infections mostly occurred in patients over 60 years old, affecting more men than women. The respiratory and transplantation department had higher incidences of deep fungal infections, and the most common sites were the respiratory and central nervous system. Candida is the main pathogen, with *Candida albicans* accounting for 60.53%, followed by *Candida glabrata* (21.05%). Patients with deep fungal infections with renal insufficiency and neurological diseases have a worse prognosis. These findings will provide references for the diagnosis and treatment of deep fungal infections.

## Data Availability Statement

The raw data supporting the conclusions of this article will be made available by the authors, without undue reservation.

## Author Contributions

S-RW, Y-KC, T-XD, and Y-YL collected and filtered the data. Z-HY and S-RW analyzed the data and wrote the initial draft of the article. Z-HY, S-RW, Y-QK, and H-BL interpreted the data. Y-QK and H-BL conceived and designed the study and critically revised the manuscript. All authors contributed to the article and approved the submitted version.

## Funding

This study was supported by the Yunnan Provincial Department of Education Science Research Fund Project, Yunnan, China (2018JS211), the Joint Special Fund of Science and Technology Department of Yunnan Province - Kunming Medical University, Yunnan, China (202001AY0700001-302), and the Key Plan Project of Science and Technology from the Department of Science and Technology of Yunnan Province (202101AY070001-022).

## Conflict of Interest

The authors declare that the research was conducted in the absence of any commercial or financial relationships that could be construed as a potential conflict of interest.

## Publisher's Note

All claims expressed in this article are solely those of the authors and do not necessarily represent those of their affiliated organizations, or those of the publisher, the editors and the reviewers. Any product that may be evaluated in this article, or claim that may be made by its manufacturer, is not guaranteed or endorsed by the publisher.
